# SREF: Semantics-Refined Feature Extraction for Long-Term Visual Localization

**DOI:** 10.3390/jimaging12020085

**Published:** 2026-02-18

**Authors:** Danfeng Wu, Kaifeng Zhu, Heng Shi, Fenfen Zhou, Minchi Kuang

**Affiliations:** 1 Beijing Key Laboratory of Information Service Engineering, Beijing Union University, Beijing 100101, China; jqrdanfeng@buu.edu.cn (D.W.); 20231083510913@buu.edu.cn (K.Z.); jqrfenfen@buu.edu.cn (F.Z.); 2 College of Robotics, Beijing Union University, Beijing 100101, China; 3 Department of Precision Instrument, Tsinghua University, Beijing 100084, China; kuangmc@mail.tsinghua.edu.cn

**Keywords:** feature extraction, fine-grained semantic, long term localization, deep learning

## Abstract

Accurate and robust visual localization under changing environments remains a fundamental challenge in autonomous driving and mobile robotics. Traditional handcrafted features often degrade under long-term illumination and viewpoint variations, while recent CNN-based methods, although more robust, typically rely on coarse semantic cues and remain vulnerable to dynamic objects. In this paper, we propose a fine-grained semantics-guided feature extraction framework that adaptively selects stable keypoints while suppressing dynamic disturbances. A fine-grained semantic refinement module subdivides coarse semantic categories into stability-homogeneous sub-classes, and a dual-attention mechanism enhances local repeatability and semantic consistency. By integrating physical priors with self-supervised clustering, the proposed framework learns discriminative and reliable feature representations. Extensive experiments on the Aachen and RobotCar-Seasons benchmarks demonstrate that the proposed approach achieves state-of-the-art accuracy and robustness while maintaining real-time efficiency, effectively bridging coarse semantic guidance with fine-grained stability estimation. Quantitatively, our method achieves strong localization performance on Aachen (up to 88.1% at night under the $\left(0.2^{{}^{\circ}},0.25\;m\right)$ threshold) and on RobotCar-Seasons (up to 57.2%/28.4% under the same threshold for day/night), demonstrating improved robustness to seasonal and illumination changes.

## 1. Introduction

Visual localization serves as a fundamental component of SLAM (simultaneous localization and mapping), enabling autonomous driving and mobile robotics to navigate and map unknown environments in real time. A typical visual SLAM pipeline consists of three stages: feature extraction, feature matching, and pose estimation, where the accuracy of pose estimation heavily depends on the quality of extracted keypoints in the first stage.

Traditional feature extraction methods, such as Harris [1], FAST (Features from Accelerated Segment Test) [2], SIFT (Scale-Invariant Feature Transform) [3], and ORB (Oriented FAST and Rotated BRIEF) [4], have long dominated this pipeline due to their simplicity and efficiency. Recent advances in deep learning have shown that convolutional neural networks can outperform handcrafted techniques in both keypoint detection [5,6] and descriptor generation [7,8], exhibiting greater resilience to appearance variations and implicitly learning invariances beyond gradient statistics. More recently, several works in semantic SLAM, such as SemanticFusion [9], DS-SLAM [10], and DynaSLAM [11], have incorporated semantic information into localization, aiming to suppress dynamic regions and emphasize semantically meaningful structures.

Despite this progress, challenges remain in achieving robust feature extraction for localization. Traditional handcrafted methods, though efficient, are highly sensitive to illumination changes, viewpoint variations, and dynamic environments. CNN-based approaches alleviate some of these issues but often treat all feature responses uniformly, failing to distinguish between stable cues and unstable details. Semantic SLAM methods further improve robustness by masking dynamic objects, yet their reliance on coarse class-level segmentation prevents finer discrimination within the same semantic region, leaving residual noise to propagate into pose estimation. These limitations highlight the need for a more adaptive mechanism to model feature reliability at a finer granularity.

To address the limitations of CNN-based approaches in distinguishing stable from unstable visual cues, we introduce an attention-driven feature extraction framework. By assigning weights to different feature elements, they enhance the robustness and discriminative power of feature extraction, making them particularly effective in complex and dynamic scenarios. First, a BAM (Bottleneck Attention Module) [12] is applied during the early feature extraction stage to emphasize locally stable keypoints and suppress noisy responses from dynamic regions. Subsequently, a CBAM (Convolutional Block Attention Module) [13] is introduced within the branching process, where it simultaneously refines feature extraction and descriptor generation by adaptively modeling both spatial and channel-wise importance. This dual-attention design produces a more discriminative and reliable descriptor set, directly improving pose estimation accuracy while maintaining adaptability across diverse environmental conditions.

To overcome the limitations of semantic-guided methods that rely on coarse, class-level stability modeling [9,10,11,14], we further propose a fine-grained semantic refinement mechanism. This approach subdivides broad semantic classes into smaller, stability-homogeneous sub-clusters, enabling more accurate suppression of unstable regions such as reflective windows or vegetation while preserving robust structural cues. In addition, a lightweight, rule-based physical prior is incorporated: objects closer to the ground plane tend to be more stable than elevated or suspended structures, consistent with the observation that “lower-layer structures act as a foundation” [15]. Rather than producing an explicit semantic map, these cues are implicitly embedded into the feature extraction process. The network directly outputs keypoints and descriptors enriched with fine-grained stability information, yielding a more discriminative representation while avoiding the computational overhead of dense semantic modeling. Our quantitative analysis confirms that this integration maintains high inference speeds and a low memory footprint, making it suitable for real-time applications. This implicit integration ensures that our method remains lightweight, yet significantly improves the robustness of feature extraction in dynamic and cluttered environments.

The main contributions of this paper are as follows:

1. We propose a physically guided, fine-grained stability modeling framework that dynamically adapts feature learning across semantic and geometric variations, surpassing prior static weighting schemes.
2. We introduce a dual-attention architecture to enhance keypoint stability and descriptor consistency in dynamic environments.
3. We embed semantic cues implicitly into the feature extraction pipeline, achieving robust and real-time localization without explicit segmentation.

The remainder of this article is organized as follows: Section 1 reviews related work on feature detection and description. Section 2 describes our proposed methodology in detail. Section 3 presents extensive experiments and analysis. Section 4 discusses current limitations and outlines future research directions, and finally, Section 5 concludes the paper.

## 2. Related Work

This section reviews prior research relevant to our work, including traditional handcrafted features, learning-based CNN approaches, and semantic-guided feature extraction methods, followed by a discussion of current limitations that motivate our design.

### 2.1. Traditional Feature Extraction

Traditional feature extraction methods form the foundation of visual SLAM and odometry, where image-based techniques estimate camera motion by detecting and matching keypoints across frames. Early detectors such as Harris [1], FAST [2], and Shi–Tomasi “good features to track” [16] focus on corners and edges that provide strong local gradients and discriminative patterns, yet they remain sensitive to viewpoint and illumination changes. Subsequent descriptors like SURF (Speeded-Up Robust Features) [17] and ORB [4] improved robustness through rotation- and scale-invariant encoding, coupling detection and description into a unified pipeline. These handcrafted methods offer high computational efficiency and repeatability, and they have long served as standard baselines in classical SLAM and visual odometry systems. However, their reliance on gradient-based heuristics limits generalization to complex real-world environments involving dynamic motion, lighting variations, or textureless regions.

### 2.2. Learning-Based Feature Extraction (CNN)

Learning-based approaches, particularly those based on convolutional neural networks (CNNs), have demonstrated superior robustness and generalization compared with handcrafted methods. CNN-based detectors such as SIPs [5] learn repeatable keypoints through probabilistic objectives, while D2D [18] reuses descriptor confidences to guide detection, improving localization accuracy without extra supervision. Descriptor learning methods, including AffNet [7], ContextDesc [19], and L2-Net [20], further enhance discriminability and invariance via architectural and loss-function innovations. Unified joint detection–description frameworks like SuperPoint [21], D2Net [22], ASLFeat [23], R2D2 [24], and DISK [25] integrate both stages end-to-end, achieving high consistency between detection and matching. XFeat [26] further optimizes this design by balancing accuracy and efficiency through a lightweight CNN architecture, enabling real-time and hardware-independent feature extraction. Recently, DarkFeat [27] extends CNN-based feature extraction to extreme low-light conditions by directly detecting and describing features from RAW images, achieving state-of-the-art robustness and a 70% reduction in computational cost. Despite these advances, most CNN-based pipelines emphasize per-frame repeatability and neglect long-term temporal or physical consistency, often treating all features equally without distinguishing stable cues from dynamic noise.

### 2.3. Semantic-Guided Feature Extraction

Beyond purely visual cues, recent research introduces semantic information to enhance robustness in dynamic or perceptually ambiguous scenes. Semantic-aware descriptors [28] embed contextual information into compact representations for improved matching consistency. LLN [29] identifies discriminative landmarks using semantic priors, while [30] leverages semantic consistency to re-rank correspondences under large appearance variations. MS^2^DG-Net [31] further exploits sparse semantic similarities between paired images to dynamically construct semantic graphs, capturing local topological relationships while maintaining permutation equivariance. Long-term localization research [32] highlights the importance of semantic stability for scene understanding and place recognition in changing environments. Recent work [33] combines semantic and depth information through domain-adaptive feature learning with Generative Adversarial Networks, effectively coupling semantic–depth cues and sequence continuity for more robust long-term visual place recognition. Building on this direction, SFD2 [14] models class-level semantic stability to balance robustness and computational efficiency. Nevertheless, most semantic-guided methods remain coarse in granularity, using fixed category weights that overlook intra-class variations and lack adaptation to physical constraints such as ground contact or structural support.

### 2.4. Compact Comparison

Table 1 summarizes key limitations of representative method categories and explicitly positions the contribution of the proposed approach with respect to the identified research gap.

The Table 1 highlights that while prior methods have addressed aspects of robustness (e.g., improved descriptors or coarse semantic masking), none simultaneously operate at fine-grained pixel-level semantics, explicitly quantify temporal photometric stability, and incorporate lightweight physical priors to guide stability weighting. Our method fills this gap by (i) deriving fine-grained sub-classes via pixel-level semantic clustering, (ii) measuring temporal photometric stability per sub-class for stability-weighted losses, and (iii) combining these with simple physical priors (e.g., ground proximity) to improve long-term localization robustness.

## 3. Method

**Figure 1:**
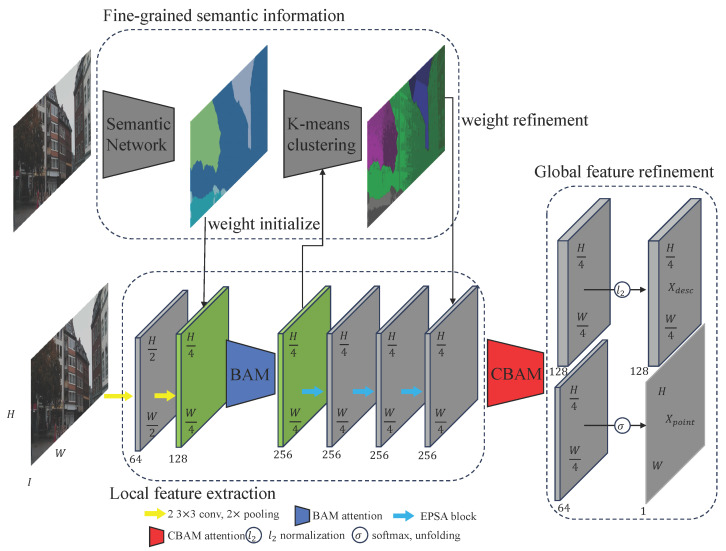
Overview of the proposed network architecture. The model integrates semantic understanding and stability estimation through two stages: coarse semantic extraction with physics-aware priors, and fine-grained weighting guided by CBAM (Convolutional Block Attention Module) during keypoint and descriptor generation. This design allows the network to adaptively emphasize semantically stable and physically consistent regions.

Our method proposes a semantics-guided local feature framework that integrates fine-grained semantic refinement, stability-aware detection, and semantically-refined descriptors enhanced by dual attention modules (Figure 1). Given an input image, a CNN backbone extracts dense features, which are refined via self-supervised semantic clustering. These semantics guide a detection branch that combines repeatability and stability to generate a reliability map, and a descriptor branch that fuses geometric–appearance features with semantic embeddings and attention mechanisms for more discriminative and consistent representations.

### 3.1. Semantic Refinement

#### 3.1.1. Semantic Clustering

Fine-grained segmentation extends a standard semantic-segmentation CNN by hierarchically subdividing each high-level class into more specific sub-classes, producing a coarse-to-fine label map. Unlike conventional approaches that assign uniform reliability to all pixels within a semantic class, our method enables pixel-level differentiation via self-supervised clustering.

Given dense feature maps ${\left\{d_{n}\right\}}_{n=1}^{N}\subset R^{d}$, we apply *k*-means clustering offline *as a preprocessing step* to obtain *m* semantic centroids $C=\left[c_{1},\ldots ,c_{m}\right]\in R^{d\times m}$ and corresponding one-hot assignments $y_{n}\in {\left\{0,1\right\}}^{m}$. The clustering objective explicitly minimizes the total within-cluster variance:

(1) $$\min_{C,\{y_{n}\}}\frac{1}{N}\sum_{n=1}^{N}\sum_{j=1}^{m}y_{n,j}{∥d_{n}-c_{j}∥}_{2}^{2},\;s.t.\;y_{n,j}\in \left\{0,1\right\},\;\sum_{j=1}^{m}y_{n,j}=1.$$

This standard *k*-means formulation minimizes the within-cluster sum of squared distances (WCSS), driving each descriptor $d_{n}$ toward its nearest centroid $c_{j}$. Cluster assignments $y_{n}^{*}$ and centroids $C^{*}$ are iteratively updated within the *k*-means procedure until convergence.

To maintain balanced partitions and avoid degenerate clusters, any empty cluster $c_{j}$ is reinitialized from a randomly selected non-empty centroid with Gaussian perturbation:

(2) $$c_{j}\leftarrow c_{r}+\epsilon ,\;r∼{\left\{1,\ldots ,m\right\}}_{\setminus j},\;\epsilon ∼N\left(0,\sigma^{2}I\right),$$

ensuring that all clusters contain valid pixel embeddings and preventing degenerate partitions during clustering.

Unlike image-level clustering [34], our approach operates at the pixel level and exploits 2D–2D correspondences between frames. This fine-grained clustering produces stability-homogeneous regions that suppress unreliable dynamics while enriching local descriptor diversity. The resulting centroids and assignments encode both semantic and geometric regularities, yielding descriptors that are physically meaningful and temporally consistent.

**Figure 2:**
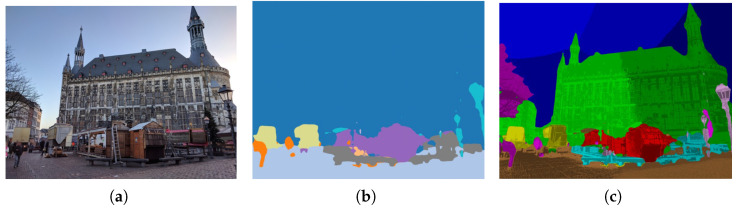
Illustration of semantic segmentation limitations in localization. (**a**) Input image. (**b**) Coarse and inconsistent region labeling from the standard ConvNeXt-based segmentation network. (**c**) Our refined segmentation with significantly richer semantics and finer spatial granularity.

Note that the clustering process is performed independently of network optimization and does not involve gradient backpropagation or online updates during training. To illustrate this effect, Figure 2 compares segmentation before and after refinement. The ConvNeXt-based semantic map (middle) captures coarse regions but exhibits inconsistent boundaries, whereas our clustering-based refinement (right) generates semantically coherent subregions, providing stronger cues for keypoint detection and descriptor learning.

#### 3.1.2. Fine-Grained Semantic Weight Allocation

To achieve robust long-term localization under dynamic and diverse conditions, we propose a fine-grained semantic weight allocation mechanism that extends beyond coarse class-based stability categories. Inspired by the physical reasoning principle in [15], our method integrates both physical plausibility and semantic granularity into the stability weighting process.

Each semantic class l∈L is first assigned a physical prior weight $w_{l}^{\left(0\right)}$ reflecting its geometric grounding and motion stability. Objects inherently anchored to the ground (e.g., roads, buildings) are given higher weights, while dynamic or floating entities (e.g., vehicles, pedestrians, sky) are penalized. The initialization is formulated as:

(3) $$w_{l}^{\left(0\right)}=\frac{1}{Z}\left(\alpha_{p}\;P_{\mathrm{ground}}\left(l\right)+\alpha_{s}\;S_{\mathrm{static}}\left(l\right)\right),$$

where $P_{\mathrm{ground}}\left(l\right)\in \left[0,1\right]$ measures the geometric contact ratio with the ground plane. $P_{\mathrm{ground}}\left(l\right)$ is implemented as a rule-based heuristic computed from the normalized vertical image coordinates. Assuming an upright camera with gravity-aligned orientation, pixels closer to the bottom of the image are assigned higher ground likelihood values, as they are physically more likely to represent grounded surfaces. $S_{\mathrm{static}}\left(l\right)\in \left[0,1\right]$ reflects the likelihood of being static, $\alpha_{p}$ and $\alpha_{s}$ are balancing factors, and *Z* normalizes the weights. This physically grounded initialization encodes the principle that “cars cannot fly,” ensuring that objects with unrealistic elevation or motion receive lower priors.

To further capture intra-class variations, each coarse semantic class *l* is decomposed into a set of fine-grained sub-classes $C_{l}=\left\{c_{1},c_{2},\ldots ,c_{n}\right\}$ obtained from the refined segmentation in Section 3.1. For each sub-class *c*, we compute a refined stability weight $w_{c}$ by integrating temporal appearance consistency $\rho_{c}$ and geometric priors:

(4) $$w_{c}={\mathrm{clip}}_{\left[0,1\right]}\;\left(w_{l}^{\left(0\right)}+\beta \;\left(\rho_{c}-{\bar{\rho }}_{l}\right)+\gamma \;\pi_{c}\right),$$

where ${\bar{\rho }}_{l}=\frac{1}{\left|Cl\right|}\sum c\in C_{l}\rho_{c}$ denotes the intra-class mean stability. The term $\pi_{c}\in \left[0,1\right]$ represents a structural stability prior assigned as a fixed scalar for each sub-class based on its typical physical properties (e.g., road surface > car > roof > tree crown). These scalars encode expert knowledge to regularize the self-supervised learning process. The coefficients β and γ control the influence of statistical and geometric refinements, respectively.

While these physical priors are simple heuristics, they effectively guide the model to focus on stable structures. We note that these priors assume a consistent vertical layout common in urban and suburban outdoor environments. For scenarios with non-upright camera configurations or unstructured environments (e.g., aerial or indoor scenes), these priors can be adapted by estimating the gravity direction or simply disabled by setting them to uniform values.

### 3.2. Semantics-Guided Feature Extraction

Recent work emphasizes local repeatability—the quality of a feature within a single frame. For long-term localization, we decompose reliability into two complementary terms: local repeatability and global stability.

#### 3.2.1. BAM-Based Local Feature Reliability

Local repeatability describes robustness to appearance and viewpoint changes. Learning-based detectors achieve stability either through ground-truth supervision or via descriptor discriminability. The former is usually more robust, yet real ground-truth is scarce. We treat SuperPoint [21] corners as pseudo ground-truth, where $G\in {\left[0,1\right]}^{H\times W}$ denotes the SuperPoint response map.

**Figure 3:**
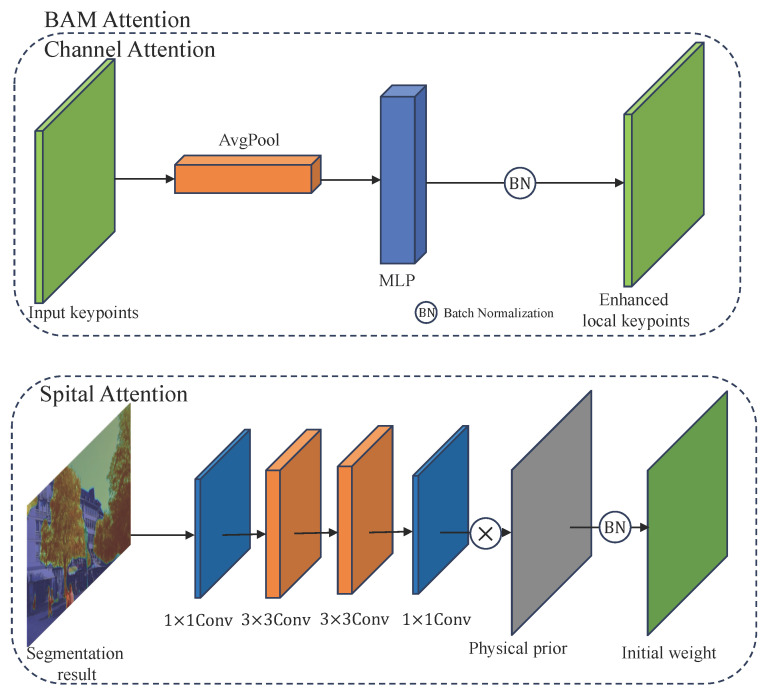
BAM (Bottleneck Attention Module) enhances local feature reliability via dual attention. Channel attention refines keypoints through pooled MLP normalization, while spatial attention fuses semantic cues and physical priors to form fine-grained stability weights.

To enhance the discriminative capacity of the reliability map, we integrate a Bottleneck Attention Module (BAM) [12]. As illustrated in Figure 3, BAM jointly models *channel* and *spatial* attention to re-weight feature responses, allowing the network to emphasize informative regions and suppress noisy ones before computing reliability. Given an intermediate feature map $F\in R^{C\times H\times W}$, the refined feature map is defined as

(5) $$F^{\prime }=F+F⊙M\left(F\right),$$

where $M\left(F\right)\in {\left[0,1\right]}^{C\times H\times W}$ is the combined attention map and ⊙ denotes element-wise multiplication. Following [12], M(F) is computed as

(6) $$M\left(F\right)=\sigma \;\left(M_{c}\left(F\right)+M_{s}\left(F\right)\right),$$

where σ is the sigmoid activation, $M_{c}\left(F\right)$ and $M_{s}\left(F\right)$ represent the channel and spatial attention maps, respectively.

Each channel of *F* encodes a particular semantic response. We first apply global average pooling to obtain a channel descriptor $F_{c}=\mathrm{AvgPool}\left(F\right)\in R^{C\times 1\times 1}$. To model inter-channel dependencies, BAM employs a two-layer MLP with reduction ratio *r*:

(7) $$M_{c}\left(F\right)=\mathrm{BN}\;\left(W_{1}\;\delta \left(W_{0}\;F_{c}+b_{0}\right)+b_{1}\right),$$

where $W_{0}\in R^{C/r\times C}$, $W_{1}\in R^{C\times C/r}$, $b_{0},b_{1}$ are biases, δ is ReLU (Rectified Linear Unit), and BN denotes batch normalization to align the scale with the spatial branch.

To capture long-range contextual dependencies, the spatial attention branch aggregates spatial cues using dilated convolutions. The feature map *F* is first reduced along channels via 1×1 convolution ($f_{1\times 1}^{0}$) and then processed by two 3×3 dilated convolutions ($f_{3\times 3}^{1},\;f_{3\times 3}^{2}$) followed by another 1×1 projection:

(8) $$M_{s}\left(F\right)=\mathrm{BN}\;(f_{1\times 1}^{3}\left(f_{3\times 3}^{2}\left(f_{3\times 3}^{1}\left(f_{1\times 1}^{0}\left(F\right)\right)\right)\right)),$$

where the dilated convolutions expand the receptive field, enabling the module to infer more context-aware spatial attention.

The final BAM-enhanced feature is then used to compute the refined local reliability:

(9) $$S_{\mathrm{rel}}\left(p\right)=\sigma \left(\lambda ,\mathrm{Conv}(F^{\prime }\left(p\right))\right),$$

where λ scales the output to [0.05,0.95] to prevent gradient saturation. This formulation ensures that locally reliable features are emphasized while unstable responses in dynamic regions are suppressed, yielding more robust keypoint detection for long-term localization.

#### 3.2.2. CBAM-Based Global Stability Estimation

**Figure 4:**
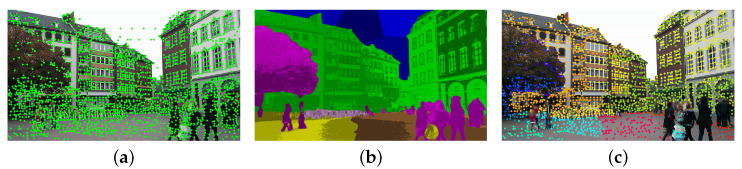
Semantics-guided feature extraction and stability-aware keypoint selection. The different colored zones in (**c**) denote the fine-grained semantic categories used to guide the filtering process. (**a**) Keypoints extracted by SPP (SuperPoint) [21], where features are densely detected but include points from unstable or dynamic regions. (**b**) Fine-grained semantic segmentation result, which provides pixel-level stability cues and highlights potentially unstable regions. (**c**) Keypoints produced by our method after semantic and stability filtering, where keypoints in unstable regions are suppressed, resulting in more reliable and semantically consistent features for long-term localization.

Global stability builds upon the semantic class of a pixel. While previous work [14] has made notable progress by partitioning the 120 ADE20K classes into four stability categories, this coarse taxonomy often fails to capture intra-class variability. For instance, tree branches may swing under strong winds and become unstable, yet they are still classified as part of a generally stable class. To address such inconsistencies, we exploit the fine-grained sub-classes obtained in the previous section, subdividing each coarse class into stability-homogeneous regions and adapting their weights instead of relying on fixed values, as illustrated in Figure 4.

**Figure 5:**
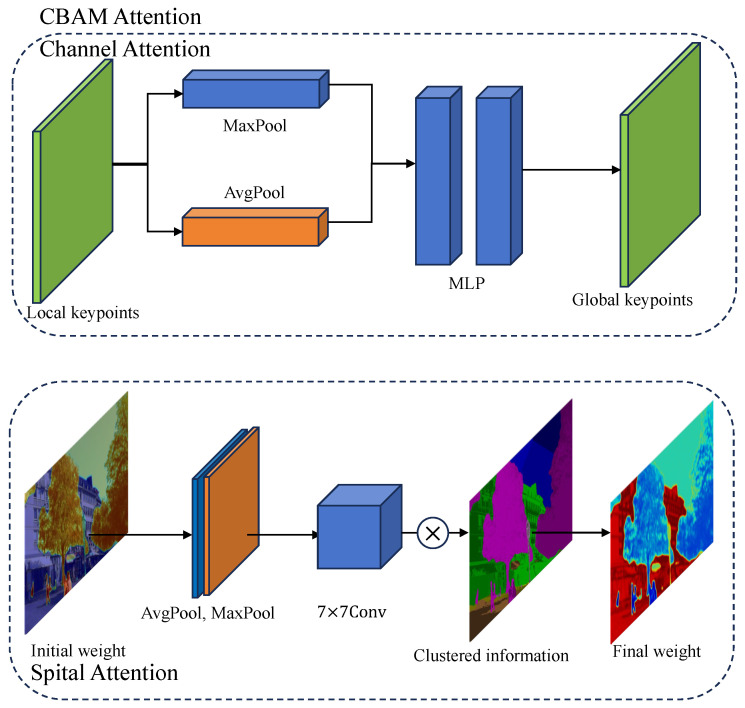
CBAM (Convolutional Block Attention Module) refines global stability estimation via dual attention. Channel attention branch aggregates local keypoints through average and max pooling followed by two-layer MLPs (Multilayer Perceptron) to generate global keypoints. Spatial attention branch fuses the pooled features and fine-grained semantic cues via a 7 × 7 convolution to produce the final stability-aware weighting map.

To further suppress instability and emphasize semantically reliable regions, we integrate a Convolutional Block Attention Module (CBAM) [13] into the global stability branch, as shown in Figure 5. Let $F_{g}\in R^{H\times W\times D}$ denote the backbone features used by the global-stability module. CBAM computes channel and spatial attention maps as follows.

The channel attention is computed by global pooling followed by a shared MLP and sigmoid activation:

(10) $$M_{c}\left(F_{g}\right)=\sigma \;\left(\mathrm{MLP}\left({\mathrm{AvgPool}}_{c}\left(F_{g}\right)\right)+\mathrm{MLP}\left({\mathrm{MaxPool}}_{c}\left(F_{g}\right)\right)\right)\in R^{D},$$

where ${\mathrm{AvgPool}}_{c}$ and ${\mathrm{MaxPool}}_{c}$ denote global average/max pooling over spatial dimensions and σ is the sigmoid.

The feature map is then channel-reweighted:

(11) $$F_{g}^{\prime }\left(p\right)=F_{g}\left(p\right)⊙M_{c}\left(F_{g}\right)$$

The spatial attention is computed from channel-pooled feature maps and a small convolutional module:

(12) $$M_{s}\left(F_{g}^{\prime }\right)=\sigma \;\left(\mathrm{Conv}\left([{\mathrm{AvgPool}}_{s}\left(F_{g}^{\prime }\right);\;{\mathrm{MaxPool}}_{s}\left(F_{g}^{\prime }\right)]\right)\right)\in R^{H\times W},$$

where [·;·] denotes channel-wise concatenation of the pooled maps. The resulting spatial attention scalar at pixel *p* is

(13) $$A_{s}\left(p\right)=M_{s}\left(F_{g}^{\prime }\right)\left(p\right)\in \left(0,1\right).$$

Intuitively, $A_{s}\left(p\right)$ highlights spatial locations that are both channel-informative and spatially consistent according to CBAM. We use this spatial attention as a multiplicative refinement of the data-driven/physics-driven stability weight $w_{c}$ (defined below). Formally, let L be the set of coarse semantic labels and $C_{l}$ the sub-classes of coarse class *l*. For each sub-class *c*, define the empirical stability ratio

(14) $$\rho_{c}=\frac{N_{c}^{\mathrm{stable}}}{N_{c}},$$

where $N_{c}$ denotes the total number of pixels belonging to sub-class *c*, and $N_{c}^{\mathrm{stable}}$ denotes the number of pixels whose photometric error over a temporal validation set is below the threshold τ. We further introduce a physical prior $\pi_{c}\in \left[0,1\right]$, with higher values assigned to grounded sub-classes. Based on these quantities, we first construct a base sub-class weight:

(15) $$w_{c}=w_{l}+\beta \;\left(\rho_{c}-{\bar{\rho }}_{l}\right)+\gamma \;\pi_{c},\;{\bar{\rho }}_{l}=\frac{1}{|C_{l}|}\sum_{c\in C_{l}}\rho_{c},$$

and clip $w_{c}$ to [0,1].

Finally, CBAM’s spatial attention modulates this base weight at pixel level. The pixel-wise global stability is defined as

(16) $$S_{\mathrm{sta}}\left(p\right)={\mathrm{clip}}_{\left[0,1\right]}\;\left(w_{c_{p}}\cdot \left(\left(1-\lambda \right)+\lambda \;A_{s}\left(p\right)\right)\right),$$

where $c_{p}$ is the sub-class of pixel *p*, λ∈[0,1] controls the influence of CBAM-driven spatial attention, and ${\mathrm{clip}}_{\left[0,1\right]}$ enforces the final stability to lie in [0,1].

### 3.3. Loss Function with Fine-Grained Semantic Weighting

Our training objective integrates semantic-guided detection and descriptor learning under the proposed fine-grained stability weighting scheme. We first describe the sub-losses and then present the unified objective function.

Detection Loss. The detection branch aims to learn reliable and repeatable keypoints. Given the predicted reliability map *S* and the target map $S_{gt}$ (Section 3.2), the detection loss is defined as:

(17) $$L_{\det}=\mathrm{BCE}\left(S,S_{gt}\right),$$

where BCE(·) denotes binary cross-entropy.

Descriptor Loss. Following prior work [14], we adopt a descriptor loss that jointly optimizes intra-class compactness and inter-class separability. This formulation remains consistent with previous studies; our contribution lies in the integration of fine-grained semantic weighting rather than the loss structure itself. Formally, the descriptor loss for pixel *p* is expressed as:

(18) $$L_{\mathrm{desc}}\left(p\right)=L_{\mathrm{intra}}\left(p\right)+L_{\mathrm{inter}}\left(p\right).$$

The intra-class loss $L_{\mathrm{intra}}$ encourages feature compactness within the same semantic subclass while preserving local diversity. Following the soft-ranking formulation in [14], it is defined as:

(19) $$L_{\mathrm{intra}}\left(p\right)=1-\mathrm{AP}\left(d_{p},S_{p}^{+}\right),$$

where $d_{p}$ denotes the descriptor at pixel *p*, $S_{p}^{+}$ is the set of positive samples sharing the same semantic label, and AP(·) measures average precision in retrieval.

The inter-class loss $L_{\mathrm{inter}}$ enhances feature separability across different semantic categories and follows the margin-based triplet structure of [14]:

(20) $$L_{\mathrm{inter}}\left(p\right)=\frac{1}{N_{p}}\;\sum_{\left(j,k\right)}{\left[∥d_{p}-d_{j}^{+}{∥}_{2}^{2}-∥d_{p}-d_{k}^{-}{∥}_{2}^{2}+m\right]}_{+},$$

where $d_{j}^{+}$ and $d_{k}^{-}$ are positive and negative samples, *m* is the margin, ${\left[\cdot \right]}_{+}=\max\left(\cdot ,0\right)$ denotes the hinge operator, and $N_{p}$ is the number of valid triplets.

Fine-Grained Semantic Weighting. Each pixel *p* is associated with a semantic–physical stability weight $w_{c_{p}}\;\in \;\left[0,1\right]$, derived from physical priors, temporal consistency, and structural depth cues (Section 3.1.2). These weights modulate the contribution of each sample in the loss function, assigning higher importance to geometrically stable and semantically reliable regions while suppressing gradients from dynamic or uncertain areas.

Overall loss. The total training loss combines detection and descriptor objectives under the proposed fine-grained weighting:

(21) $$L_{loss}=\sum_{p}w_{c_{p}}\left(L_{\det}\left(p\right)+\lambda \left(L_{\mathrm{intra}}\left(p\right)+L_{\mathrm{inter}}\left(p\right)\right)\right),$$

where λ balances the descriptor and detection branches.

## 4. Experiments

We first give implementation details. Then, we test our method on visual localization tasks in Section 4.1 and analyze the running time in Section 4.2. Finally, we perform an ablation study in Section 4.3.

Implementation Details. The training set comprises reference images from the Aachen v1.0 dataset together with additional web-collected images. As SFD2 [14] and R2D2 [24], we augment the training images via style transfer. To alleviate segmentation noise introduced by stylization, semantic labels for the stylized samples are inferred from their corresponding original images. The network is developed in PyTorch (>=1.8) and optimized with the Adam solver ($\beta_{1}$ = 0.9, $\beta_{2}$ = 0.9). Training is performed on one RTX 4090 GPU for 40 epochs with a mini-batch size of 4 and a weight-decay coefficient of $4\times 10^{-4}$.

### 4.1. Localization Test

We tested our method on Aachen (v1.0 and v1.1) and RobotCar-Seasons datasets under various illumination, season, and weather conditions. Aachen v1.0 contains 4328 reference and 922 query images captured around the Aachen city center. Aachen v1.1 expands v1.0 by adding 2369 reference and 93 query images. RoCaS has 26,121 reference and 11,934 query images. It is challenging because of various conditions of day query images and poor lighting of night query images in suburban areas. Following the evaluation protocol of the long-term visual localization benchmark [35], we report the success ratio at the error thresholds of (2°,0.25 m), (5°,0.5 m), (10°,5 m) as metric.

Baselines. We evaluate our model against recent methods that leverage semantic information, including LLN [14,29,31,33,36,37,38,39], as well as approaches based on learned feature representations [3,21,22,23,24,26,40,41]. Following established protocols, we employ the HLoc pipeline for 3D reconstruction and mutual nearest neighbor (MNN) matching. Furthermore, we compare our approach with state-of-the-art learned matchers [42,43,44,45,46,47,48,49]. All reported results are taken either from the visual benchmark [35] or the original publications.

Quantitative Results. Table 2, Table 3 and Table 4 present the localization accuracy on the Aachen (v1.0/v1.1) and RobotCar-Seasons benchmarks. Overall, our method consistently achieves the best or on-par performance across all datasets and conditions, particularly under challenging night and weather scenarios.

Comparison with semantic-based methods (S). Among methods explicitly leveraging semantic cues, LBR and SFD2 exhibit strong baselines due to their use of building-instance matching and stability-driven semantic weighting, respectively. While SFD2 assigns stability through a static, class-based mapping, our method learns a dynamic fine-grained stability field that adapts to intra-class variations and physical scene constraints. Built upon this principle, we introduce a fine-grained semantic weight allocation mechanism that refines class-level stability into sub-class semantics guided by geometric priors. This adaptive design enables the model to better capture subtle variations in motion, structure, and illumination, effectively suppressing unstable regions such as vegetation or reflective surfaces. As shown in Table 2, our method slightly surpasses SFD2 in both day and night conditions on Aachen v1.0 and achieves consistent gains on Aachen v1.1 and RobotCar-Seasons, demonstrating improved robustness in dynamic and low-light environments.

Comparison with learned feature methods (L). Compared with learned feature extractors such as D2Net, R2D2, and ASLFeat achieve higher accuracy thanks to end-to-end descriptor optimization. However, these methods still struggle under illumination changes and dynamic interference. Our semantics-guided model yields superior accuracy and maintains high stability at night, outperforming all learned counterparts. The results confirm that incorporating fine-grained semantic priors within feature extraction effectively enhances descriptor discriminability and localization reliability.

Comparison with Advanced matchers (M). Modern matching frameworks like SuperGlue [42], SGMNet [43], and ClusterGNN [44] improve correspondence consistency through graph reasoning or attention-based matching. Nevertheless, they often rely on computationally intensive post-matching modules. In contrast, our method achieves comparable or better performance using only simple mutual nearest neighbor (MNN) matching, validating that the proposed fine-grained semantic refinement and attention-based feature extraction already yield highly distinctive and geometrically stable features.

**Figure 6:**
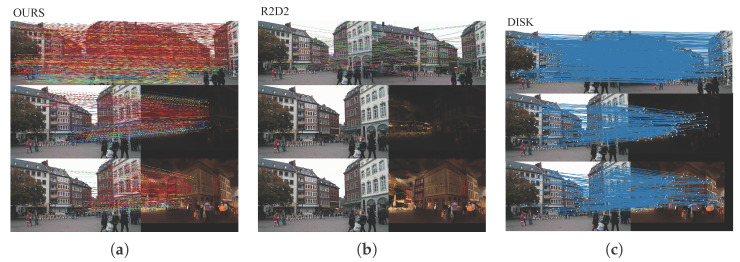
Comparison of feature matching performance under challenging conditions. (**a**) Our proposed method; (**b**) R2D2 [24]; (**c**) DISK [25]. Our model provides more reliable inliers and denser correspondences compared to other learned features.

As shown in Figure 6, our method achieves more accurate and denser feature correspondences compared to R2D2 and DISK, even under challenging day–night variations. While R2D2 and DISK rely on SuperGlue for correspondence estimation, they still suffer from sparse or unstable matches—R2D2 produces few correspondences in low-light scenes, whereas DISK yields more but less reliable ones. In contrast, our approach directly establishes correspondences using a nearest-neighbor matching strategy, enabled by semantically weighted feature extraction and fine-grained stability refinement. This design effectively suppresses unstable regions while preserving semantically consistent structures, leading to higher inlier ratios and improved robustness under illumination and nighttime conditions.

### 4.2. Visualization and Effectiveness of Fine-Grained Semantic Weighting

**Figure 7:**
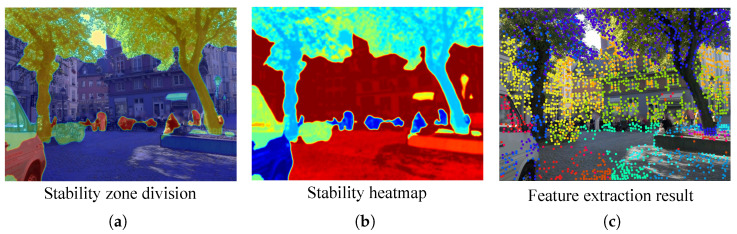
Visualization of the proposed fine-grained semantic weights and dual-attention responses. The different colored zones in (**a**) represent the clustered fine-grained semantic regions, while the colored dots in (**c**) visualize the extracted feature points where the colors reflect the semantic refinement and stability responses derived from (**b**). (**a**) Fine-grained semantic zone division obtained from self-supervised clustering; (**b**) stability weight heatmap where red represents high stability and blue represents low weight; (**c**) resulting feature extraction map. The weighting mechanism effectively emphasizes physically stable and semantically consistent regions while suppressing dynamic or uncertain areas.

To further validate the effectiveness of the proposed fine-grained semantic weighting mechanism, we visualize the stability-aware segmentation and the corresponding weight heatmap, as shown in Figure 7. The visualization consists of three parts: (i) the fine-grained semantic zone division obtained from self-supervised clustering, (ii) the computed stability heatmap based on semantic, geometric, and physical priors, and (iii) the resulting feature extraction map after applying the proposed weighting.

As observed, the fine-grained segmentation produces semantically coherent yet physically distinct regions, which allow the weighting process to better capture intra-class variations in stability. The stability heatmap assigns higher weights to geometrically grounded and static areas such as roads, walls, and building structures, while suppressing dynamic or weakly constrained regions. This adaptive weighting helps the network focus on physically reliable cues during training, leading to improved consistency in keypoint detection and descriptor learning, particularly under dynamic and low-light scenarios.

Quantitatively, this fine-grained weighting yields more stable localization performance across both static and dynamic sequences, demonstrating the advantage of incorporating physical priors and intra-class semantic distinctions into the feature extraction pipeline.

### 4.3. Ablation Study

Table 5 presents the ablation results on the Aachen_v1.1 dataset. Starting from the baseline with semantic weighting (SW), introducing the proposed fine-grained weighting (FW) leads to consistent improvements by modeling intra-class stability variations and integrating physical priors. Adding the dual attention (DA) module further enhances robustness by jointly refining spatial and channel-wise cues, allowing the network to better emphasize physically stable regions and suppress transient or ambiguous features. Together, SW, FW, and DA achieve the best performance, improving both day and night localization success ratios, especially under challenging illumination and dynamic conditions.

Table 6 further explores the impact of clustering granularity on the effectiveness of fine-grained weighting by varying the number of clusters *k* for each semantic class. Increasing *k* from 2 to 4 leads to a significant performance boost, particularly in night-time queries. This demonstrates that finer subdivisions allow the stability weights to better distinguish between reliable structural elements and unstable dynamic parts within the same semantic category. However, further increasing *k* to 5 results in marginal performance saturation, suggesting that overly fragmented subdivisions may introduce noise or redundant spatial constraints.

Notably, since our clustering is performed as an offline pre-processing step based on spatial coordinates, it introduces zero computational overhead during the training phase while providing stable and consistent geometric guidance. Consequently, k=4 is chosen as our default configuration to balance structural granularity and training stability.

## 5. Limitations and Future Work

Despite the promising results achieved in urban long-term localization, several limitations of the proposed framework remain to be addressed in future research.

### 5.1. Offline Clustering vs. Online Adaptation

A primary constraint of the current implementation is that the fine-grained semantic sub-classes are generated via an offline k-means clustering process prior to training. While this approach ensures training stability and computational efficiency, it prevents the sub-class boundaries from adapting to the evolving feature space of the encoder during the self-supervised learning process. Future work will explore the integration of online or incremental clustering mechanisms to allow the semantic subdivisions to co-evolve with the feature representations.

### 5.2. Scope of Generalization

Our experimental evaluation has been primarily focused on urban driving scenarios (Aachen and RobotCar-Seasons), which are characterized by gravity-aligned camera orientations and consistent vertical scene layouts. The performance of the proposed physical priors, such as ground proximity, has not been explicitly validated in diverse domains like indoor environments, aerial platforms, or unstructured off-road scenes. In these scenarios, the “upright camera” assumption may not hold. We plan to extend our framework by incorporating IMU-based gravity estimation or learned spatial priors to enhance its versatility across cross-domain environments.

### 5.3. Extreme Meteorological Conditions

While our method demonstrates improved robustness to illumination and moderate seasonal shifts, its performance boundaries under more extreme weather conditions, such as dense fog, heavy snowfall, or sandstorms, have not been fully tested. The performance trend observed from the relatively clear Aachen dataset to the more challenging RobotCar-Seasons (which includes rain and light snow) suggests a potential degradation in localization precision when photometric information is severely compromised. Evaluating the model on specialized adverse-weather datasets and developing specialized stability-weighting strategies for these conditions remain key priorities for our future work.

### 5.4. Dependency on Pre-Trained Segmenters

Lastly, the quality of our fine-grained refinement depends on the initial masks provided by the semantic segmenter. In environments where the segmenter fails (e.g., highly unusual or textureless objects), the stability weights may be sub-optimal. Exploring joint learning of semantic segmentation and feature stability is a promising direction to improve the system’s end-to-end reliability.

## 6. Conclusions

In this paper, we presented a fine-grained semantics-guided feature extraction framework for robust long-term visual localization. Our approach enhances stability modeling through fine-grained semantic weight allocation, which refines coarse class-level priors using sub-class semantics and geometric constraints. By integrating dual attention mechanisms, the network effectively suppresses dynamic or unstable regions while emphasizing semantically consistent structures. This joint modeling of semantic granularity, physical priors, and attention yields features that are both geometrically distinctive and semantically stable. Extensive experiments on Aachen and RobotCar-Seasons benchmarks demonstrate that our method outperforms existing semantic and learned feature baselines, achieving superior robustness under challenging illumination, weather, and viewpoint variations while maintaining real-time performance.

## Figures and Tables

**Table 1:** Comparison of representative methods and our positioning.

Category	Key Limitations	Proposed Contribution & Gap Addressing
Handcrafted	Illum./viewpoint sensitivity; lack of semantic/temporal context.	Gap: No scene understanding. *Ours:* Robustness via semantic guidance and stability weighting.
Learning-based	Focus on patch repeatability; ignore pixel-level temporal consistency.	Gap: No long-term stability modeling. *Ours:* Self-supervised stability supervision via pixel clustering.
Semantic-aided	Coarse class-level masks; neglect intra-class heterogeneity.	Gap: Coarse granularity. *Ours:* Subdivides classes into fine-grained sub-classes via physical priors.
Ours (SREF)	Integrated Framework: Fuses fine-grained semantics, temporal stability, and physical priors.	

**Table 2:** Quantitative evaluation of localization performance on the Aachen v1.0 benchmark across day and night conditions. Precision rates (%) are reported at three standard pose error thresholds: T1 ($0.2^{{}^{\circ}}$, 0.25 m), T2 ($5^{{}^{\circ}}$, 0.5 m), and T3 ($10^{{}^{\circ}}$, 5 m). Methods are grouped into: (S) semantic-based approaches, (L) local feature-based extractors, and (M) advanced matching pipelines. Inference efficiency is quantified via frames per second (FPS) and peak GPU memory consumption (MB) to illustrate the trade-off between accuracy and resource usage.

Group	Method	*Day* (T1/T2/T3)	*Night* (T1/T2/T3)	FPS ↑	Mem. (MB) ↓
S	SSM [37]	71.8/91.5/96.8	58.2/76.5/90.8	15.2	458.6
	LLN [29]	62.4/71.8/79.9	35.7/44.9/54.1	11.1	**324.2**
	MS2DG [31]	85.7/93.9/97.9	72.4/82.7/92.9	10.5	892.5
	LBR [36]	88.3/95.6/**98.8**	84.7/93.9/100.0	18.5	645.8
	SFD2 [14]	88.2/96.0/98.7	87.8/94.9/100.0	**30.1**	1184.3
	**OURS**	**88.8/96.4**/98.5	**88.1/95.0/100.0**	24.6	584.2
L	SPP [21]	80.5/87.4/94.2	42.9/62.2/76.5	**76.3**	412.7
	D2Net [22]	84.8/92.6/97.5	84.7/90.8/96.9	2.4	1846.5
	XFeat [26]	84.7/91.5/96.5	77.6/89.8/98.0	55.0	**286.3**
	R2D2 [24]	-	76.5/90.8/100.0	13.8	1422.0
	PoSFeat [41]	-	81.6/90.8/100.0	8.4	954.1
	ASLFeat [23]	-	81.6/87.8/100.0	8.9	1104.2
	**OURS**	**88.8/96.4/98.5**	**88.1/95.0/100.0**	24.6	584.2
M	DeViLoc [49]	87.4/94.8/98.2	87.8/93.9/100.0	1.2	3215.7
	MakeGNN [50]	83.9/91.3/95.6	66.3/75.5/87.8	4.5	2108.4
	MSGA [48]	83.4/92.0/96.7	66.3/89.8/96.6	3.8	2412.3
	S2DNet [51]	84.5/90.3/95.3	74.5/82.7/94.9	2.1	2806.5
	NCMNet [47]	84.2/92.5/96.0	48.2/59.7/75.4	6.4	1910.8
	SPP+SPG [21,42]	**89.6**/95.4/98.8	86.7/93.9/100.0	6.8	1512.6
	SPP+SGMNet [21,43]	86.8/94.2/97.7	83.7/91.8/99.0	10.2	1208.4
	SPP+CluGNN [44]	89.4/95.5/98.5	81.6/93.9/100.0	7.1	1815.3
	R-SCoRe [52]	74.8/86.9/96.4	64.3/89.8/96.9	13.8	**52.4**
	**OURS**	88.8/**96.4**/98.5	**88.1/95.0/100.0**	**24.6**	584.2

Note: Bold values indicate the best performance; ↑ and ↓ indicate that higher and lower values are better, respectively; italics are used for environmental condition headers.

**Table 3:** Localization results on the Aachen v1.1 dataset, which contains expanded reference imagery and more challenging night-time queries compared to v1.0. Precision rates (%) are measured against standard thresholds T1–T3. This table highlights our performance relative to (L) local feature descriptors and (M) sophisticated matching-based frameworks. Inference efficiency is quantified via frames per second (FPS) and peak GPU memory consumption (MB) to illustrate the trade-off between accuracy and resource usage.

Group	Method	*Day* (T1/T2/T3)	*Night* (T1/T2/T3)	FPS ↑	Mem.(MB) ↓
L	SPP [21]	87.9/93.6/96.8	70.2/84.8/93.7	**76.1**	**414.2**
	D2Net [22]	84.1/91.0/95.5	63.4/83.8/92.1	2.3	1848.1
	R2D2 [24]	88.8/95.3/97.8	72.3/88.5/94.2	13.6	1425.4
	ASLFeat [23]	88.0/95.4/98.2	70.7/84.3/94.2	8.8	1107.5
	CAPS+SIFT [3,53]	82.4/91.3/95.9	61.3/83.8/95.3	10.4	1015.6
	PoSFeat [41]	-	73.8/87.4/98.4	8.3	958.2
	**OURS**	**89.6/96.7/99.5**	**79.4/92.8/99.9**	24.4	586.8
M	SPP+SGMNet [21,43]	88.7/96.2/98.9	75.9/89.0/99.0	10.1	1211.3
	SPP+SPG [21,42]	**89.8**/96.1/99.4	77.0/90.6/**100.0**	6.7	1516.4
	NCMNet [47]	82.6/91.9/97.2	31.4/38.7/45.5	6.3	1914.5
	LoFTR [46]	88.7/95.6/99.0	78.5/90.6/99.0	4.0	2849.2
	ASpanFormer [45]	89.4/95.6/99.0	77.5/91.6/99.5	3.4	3112.5
	**OURS**	89.6/**96.7/99.5**	**79.4/92.8**/99.9	**24.4**	**586.8**

Note: Bold values indicate the best performance; ↑ and ↓ indicate that higher and lower values are better, respectively; italics are used for environmental condition headers.

**Table 4:** Localization results on the RobotCar-Seasons dataset. Precision rates (%) are reported at thresholds T1: (0.2°, 0.25 m), T2: (5°, 0.5 m), T3: (10°, 5 m). Inference efficiency is quantified via frames per second (FPS) and peak GPU memory consumption (MB) to illustrate the trade-off between accuracy and resource usage.

Group	Method	*Day* (T1–T3)	*Night* (T1–T3)	*N-Rain* (T1–T3)	FPS ↑	Mem. (MB) ↓
S	SSM [37]	54.5/81.6/96.7	10.0/23.7/45.4	14.5/33.2/47.5	15.0	462.1
	LLN [29]	7.9/30.0/85.9	11.9/26.0/55.0	15.7/34.5/60.5	10.9	**327.5**
	DASGIL-FD [38]	8.7/30.7/81.3	1.6/4.8/19.9	1.8/4.3/21.6	14.6	514.8
	ToDayGAN [39]	52.2/80.1/95.9	16.4/43.2/73.3	24.1/50.5/74.1	8.3	1209.3
	SFD2 [14]	56.9/81.6/97.4	27.6/66.2/90.2	43.0/71.1/90.0	**29.8**	1188.6
	LBR [36]	56.7/81.7/**98.2**	24.9/62.3/86.1	**47.5/73.4**/90.0	18.2	649.2
	Method [33]	8.4/29.2/86.9	3.9/9.5/33.1	1.8/5.9/19.1	12.3	558.7
	**OURS**	**57.2/81.8**/97.2	**28.4/67.5/91.1**	44.1/71.5/**90.4**	24.2	589.5
L	SPP [21]	56.5/81.5/97.1	16.9/41.6/71.5	22.0/45.0/68.0	**75.8**	**418.3**
	D2Net [22]	54.5/80.0/95.3	18.0/39.7/53.9	22.7/40.5/56.1	2.3	1852.4
	R2D2 [24]	**57.4/81.9/97.9**	18.3/43.4/67.8	29.1/50.2/68.2	13.5	1428.1
	CAPS [53]	56.0/81.5/96.5	21.9/54.3/86.8	27.0/58.9/85.9	10.3	1018.6
	ASLFeat [23]	57.1/81.9/98.4	23.5/55.9/80.1	41.1/66.8/86.1	8.7	1110.4
	**OURS**	57.2/81.8/97.2	**28.4/67.5/91.1**	**44.1/71.5/90.4**	24.2	589.5
M	SPP+SPG [21,42]	56.9/81.7/98.1	24.2/62.6/87.4	42.3/69.3/90.2	6.6	1519.2
	DeViLoc [49]	56.9/81.8/98.0	**31.3/68.9**/92.4	43.9/**77.3**/95.5	1.1	3224.5
	Pixloc [54]	56.9/**82.0/98.1**	24.2/62.8/88.4	**45.5**/72.5/90.7	0.5	4132.6
	AHM [55]	45.7/78.0/95.1	16.2/55.3/**93.6**	28.4/68.4/**95.5**	2.7	2519.8
	**OURS**	**57.2**/81.8/97.2	28.4/67.5/91.1	44.1/71.5/90.4	**24.2**	**589.5**

Note: Bold values indicate the best performance; ↑ and ↓ indicate that higher and lower values are better, respectively; italics are used for environmental condition headers.

**Table 5:** Ablation study on Aachen_v1.1 dataset. SW: Semantic Weighting, FW: Fine-grained Weighting, DA: Dual Attention.

SW	FW	DA	Success Ratio (%)	
			*Day*	*Night*
			(0.2°, 0.25 m)/(5°, 0.5 m)/(10°, 5 m)	
	✓	✓	85.2/92.4/98.1	76.3/85.7/96.5
✓		✓	87.9/94.1/98.8	75.0/86.2/97.7
✓	✓		89.6/96.4/99.5	79.4/92.8/99.9
✓	✓	✓	**91.3**/**97.5**/**99.8**	**82.1**/**94.6**/**100.0**

Note: The check marks (✓) indicate the components selected for each experimental configuration; bold values represent the best results for each evaluation metric; italics are used for environmental condition headers.

**Table 6:** Sensitivity to the number of clusters *k* (Aachen_v1.1). Success ratios are shown for thresholds $\left(0.2^{\circ },\;0.25\;m\right)/\left(5^{\circ },\;0.5\;m\right)/\left(10^{\circ },\;5\;m\right)$.

Experiment	Setting	*Day*	*Night*
Number of clusters *k*	k=2	85.2/92.4/98.1	76.3/85.7/96.5
	k=3	89.6/96.4/99.5	79.4/92.8/99.9
	k=4	**91.3/97.5/99.8**	**82.1/94.6/100.0**
	k=5	91.0/97.3/99.7	81.8/94.3/100.0

Note: Bold values indicate the best results for each evaluation metric; italics are used for environmental condition headers.

## Data Availability

The data presented in this study are available on request from the corresponding author due to privacy restrictions.

## Abbreviations

The following abbreviations are used in this manuscript:

SLAM	Simultaneous localization and mapping
BAM	Bottleneck Attention Module
CBAM	Convolutional Block Attention Module
CNN	Convolutional Neural Network
SW	Semantic weighting
FW	Fine-grained weighting
DA	Dual attention module

## References

[1] Harris C., Stephens M. A combined corner and edge detector. Proceedings of the Alvey Vision Conference.

[2] Rosten E., Drummond T. (2006). Machine learning for high-speed corner detection. European Conference on Computer Vision.

[3] Lowe D.G. (2004). Distinctive image features from scale-invariant keypoints. Int. J. Comput. Vis..

[4] Rublee E., Rabaud V., Konolige K., Bradski G. ORB: An efficient alternative to SIFT or SURF. Proceedings of the 2011 International Conference on Computer Vision.

[5] Cieslewski T., Derpanis K.G., Scaramuzza D. Sips: Succinct interest points from unsupervised inlierness probability learning. Proceedings of the 2019 International Conference on 3D Vision (3DV).

[6] Suwanwimolkul S., Komorita S., Tasaka K. Learning of low-level feature keypoints for accurate and robust detection. Proceedings of the IEEE/CVF Winter Conference on Applications of Computer Vision.

[7] Mishkin D., Radenovic F., Matas J. Repeatability is not enough: Learning affine regions via discriminability. Proceedings of the European Conference on Computer Vision (ECCV).

[8] Dusmanu M., Miksik O., Schönberger J.L., Pollefeys M. Cross-descriptor visual localization and mapping. Proceedings of the IEEE/CVF International Conference on Computer Vision.

[9] McCormac J., Handa A., Davison A., Leutenegger S. Semanticfusion: Dense 3D semantic mapping with convolutional neural networks. Proceedings of the 2017 IEEE International Conference on Robotics and automation (ICRA).

[10] Yu C., Liu Z., Liu X.J., Xie F., Yang Y., Wei Q., Fei Q. DS-SLAM: A semantic visual SLAM towards dynamic environments. Proceedings of the 2018 IEEE/RSJ International Conference on Intelligent Robots and Systems (IROS).

[11] Bescos B., Fácil J.M., Civera J., Neira J. (2018). DynaSLAM: Tracking, mapping, and inpainting in dynamic scenes. IEEE Robot. Autom. Lett..

[12] Park J., Woo S., Lee J.Y., Kweon I.S. (2020). A simple and light-weight attention module for convolutional neural networks. Int. J. Comput. Vis..

[13] Woo S., Park J., Lee J.Y., Kweon I.S. Cbam: Convolutional block attention module. Proceedings of the European Conference on Computer Vision (ECCV).

[14] Xue F., Budvytis I., Cipolla R. Sfd2: Semantic-guided feature detection and description. Proceedings of the IEEE/CVF Conference on Computer Vision and Pattern Recognition.

[15] Choi S., Kim J.T., Choo J. Cars can’t fly up in the sky: Improving urban-scene segmentation via height-driven attention networks. Proceedings of the IEEE/CVF Conference on Computer Vision and Pattern Recognition.

[16] Shi J. Good features to track. Proceedings of the 1994 Proceedings of IEEE Conference on Computer Vision and Pattern Recognition.

[17] Bay H., Tuytelaars T., Van Gool L. (2006). Surf: Speeded up robust features. European Conference on Computer Vision.

[18] Tian Y., Balntas V., Ng T., Barroso-Laguna A., Demiris Y., Mikolajczyk K. D2D: Keypoint extraction with describe to detect approach. Proceedings of the Asian Conference on Computer Vision.

[19] Luo Z., Shen T., Zhou L., Zhang J., Yao Y., Li S., Fang T., Quan L. Contextdesc: Local descriptor augmentation with cross-modality context. Proceedings of the IEEE/CVF Conference on Computer Vision and Pattern Recognition.

[20] Tian Y., Fan B., Wu F. L2-net: Deep learning of discriminative patch descriptor in euclidean space. Proceedings of the IEEE Conference on Computer Vision and Pattern Recognition.

[21] DeTone D., Malisiewicz T., Rabinovich A. Superpoint: Self-supervised interest point detection and description. Proceedings of the IEEE Conference on Computer Vision and Pattern Recognition Workshops.

[22] Dusmanu M., Rocco I., Pajdla T., Pollefeys M., Sivic J., Torii A., Sattler T. D2-net: A trainable cnn for joint description and detection of local features. Proceedings of the IEEE/CVF Conference on Computer Vision and Pattern Recognition.

[23] Luo Z., Zhou L., Bai X., Chen H., Zhang J., Yao Y., Li S., Fang T., Quan L. Aslfeat: Learning local features of accurate shape and localization. Proceedings of the IEEE/CVF Conference on Computer Vision and Pattern Recognition.

[24] Revaud J., De Souza C., Humenberger M., Weinzaepfel P. (2019). R2d2: Reliable and repeatable detector and descriptor. Adv. Neural Inf. Process. Syst..

[25] Tyszkiewicz M., Fua P., Trulls E. (2020). Disk: Learning local features with policy gradient. Adv. Neural Inf. Process. Syst..

[26] Potje G., Cadar F., Araujo A., Martins R., Nascimento E.R. Xfeat: Accelerated features for lightweight image matching. Proceedings of the IEEE/CVF Conference on Computer Vision and Pattern Recognition.

[27] He Y., Hu Y., Zhao W., Li J., Liu Y.J., Han Y., Wen J. DarkFeat: Noise-robust feature detector and descriptor for extremely low-light RAW images. Proceedings of the AAAI Conference on Artificial Intelligence.

[28] Naseer T., Oliveira G.L., Brox T., Burgard W. Semantics-aware visual localization under challenging perceptual conditions. Proceedings of the 2017 IEEE International Conference on Robotics and Automation (ICRA).

[29] Xin Z., Cai Y., Lu T., Xing X., Cai S., Zhang J., Yang Y., Wang Y. Localizing discriminative visual landmarks for place recognition. Proceedings of the 2019 International Conference on Robotics and Automation (ICRA).

[30] Toft C., Stenborg E., Hammarstrand L., Brynte L., Pollefeys M., Sattler T., Kahl F. Semantic match consistency for long-term visual localization. Proceedings of the European Conference on Computer Vision (ECCV).

[31] Dai L., Liu Y., Ma J., Wei L., Lai T., Yang C., Chen R. MS2DG-Net: Progressive correspondence learning via multiple sparse semantics dynamic graph. Proceedings of the IEEE/CVF conference on Computer Vision and Pattern Recognition.

[32] Stenborg E., Toft C., Hammarstrand L. Long-term visual localization using semantically segmented images. Proceedings of the 2018 IEEE International Conference on Robotics and Automation (ICRA).

[33] Tan Y., Ji P., Zhang Y., Ge F., Zhu S. (2024). Learning robust representation and sequence constraint for retrieval-based long-term visual place recognition. Eng. Appl. Artif. Intell..

[34] Caron M., Bojanowski P., Joulin A., Douze M. Deep clustering for unsupervised learning of visual features. Proceedings of the European Conference on Computer Vision (ECCV).

[35] Sattler T., Maddern W., Toft C., Torii A., Hammarstrand L., Stenborg E., Safari D., Okutomi M., Pollefeys M., Sivic J. Benchmarking 6dof outdoor visual localization in changing conditions. Proceedings of the IEEE Conference on Computer Vision and Pattern Recognition.

[36] Xue F., Budvytis I., Reino D.O., Cipolla R. Efficient large-scale localization by global instance recognition. Proceedings of the IEEE/CVF Conference on Computer Vision and Pattern Recognition.

[37] Shi T., Shen S., Gao X., Zhu L. Visual localization using sparse semantic 3D map. Proceedings of the 2019 IEEE International Conference on Image Processing (ICIP).

[38] Hu H., Qiao Z., Cheng M., Liu Z., Wang H. (2020). Dasgil: Domain adaptation for semantic and geometric-aware image-based localization. IEEE Trans. Image Process..

[39] Anoosheh A., Sattler T., Timofte R., Pollefeys M., Van Gool L. Night-to-day image translation for retrieval-based localization. Proceedings of the 2019 International Conference on Robotics and Automation (ICRA).

[40] Tian Y., Yu X., Fan B., Wu F., Heijnen H., Balntas V. Sosnet: Second order similarity regularization for local descriptor learning. Proceedings of the IEEE/CVF Conference on Computer Vision and Pattern Recognition.

[41] Li K., Wang L., Liu L., Ran Q., Xu K., Guo Y. Decoupling makes weakly supervised local feature better. Proceedings of the IEEE/CVF Conference on Computer Vision and Pattern Recognition.

[42] Sarlin P.E., DeTone D., Malisiewicz T., Rabinovich A. Superglue: Learning feature matching with graph neural networks. Proceedings of the IEEE/CVF Conference on Computer Vision and Pattern Recognition.

[43] Chen H., Luo Z., Zhang J., Zhou L., Bai X., Hu Z., Tai C.L., Quan L. Learning to match features with seeded graph matching network. Proceedings of the IEEE/CVF International Conference on Computer Vision.

[44] Shi Y., Cai J.X., Shavit Y., Mu T.J., Feng W., Zhang K. Clustergnn: Cluster-based coarse-to-fine graph neural network for efficient feature matching. Proceedings of the IEEE/CVF Conference on Computer Vision and Pattern Recognition.

[45] Chen H., Luo Z., Zhou L., Tian Y., Zhen M., Fang T., Mckinnon D., Tsin Y., Quan L. (2022). Aspanformer: Detector-free image matching with adaptive span transformer. European Conference on Computer Vision.

[46] Sun J., Shen Z., Wang Y., Bao H., Zhou X. LoFTR: Detector-free local feature matching with transformers. Proceedings of the IEEE/CVF Conference on Computer Vision and Pattern Recognition.

[47] Liu X., Qin R., Yan J., Yang J. (2024). Ncmnet: Neighbor consistency mining network for two-view correspondence pruning. IEEE Trans. Pattern Anal. Mach. Intell..

[48] Gong Z., Xiao G., Shi Z., Chen R., Yu J. (2024). MSGA-Net: Progressive feature matching via multi-layer sparse graph attention. IEEE Trans. Circuits Syst. Video Technol..

[49] Giang K.T., Song S., Jo S. Learning to produce semi-dense correspondences for visual localization. Proceedings of the IEEE/CVF Conference on Computer Vision and Pattern Recognition.

[50] Li Z., Ma J. (2024). Learning feature matching via matchable keypoint-assisted graph neural network. IEEE Trans. Image Process..

[51] Germain H., Bourmaud G., Lepetit V. (2020). S2DNet: Learning image features for accurate sparse-to-dense matching. European Conference on Computer Vision.

[52] Jiang X., Wang F., Galliani S., Vogel C., Pollefeys M. R-score: Revisiting scene coordinate regression for robust large-scale visual localization. Proceedings of the Computer Vision and Pattern Recognition Conference.

[53] Wang Q., Zhou X., Hariharan B., Snavely N. (2020). Learning feature descriptors using camera pose supervision. European Conference on Computer Vision.

[54] Sarlin P.E., Unagar A., Larsson M., Germain H., Toft C., Larsson V., Pollefeys M., Lepetit V., Hammarstrand L., Kahl F. Back to the feature: Learning robust camera localization from pixels to pose. Proceedings of the IEEE/CVF Conference on Computer Vision and Pattern Recognition.

[55] Germain H., Bourmaud G., Lepetit V. Sparse-to-dense hypercolumn matching for long-term visual localization. Proceedings of the 2019 International Conference on 3D Vision (3DV).

